# Pnictogen Bonding at the Core of a Carbene-Stiborane-Gold Complex: Impact on Structure and Reactivity

**DOI:** 10.1021/acs.organomet.4c00347

**Published:** 2024-09-26

**Authors:** Paula Castro Castro, François P. Gabbaï

**Affiliations:** Department of Chemistry, Texas A&M University, College Station, Texas 77843-3255, United States

## Abstract

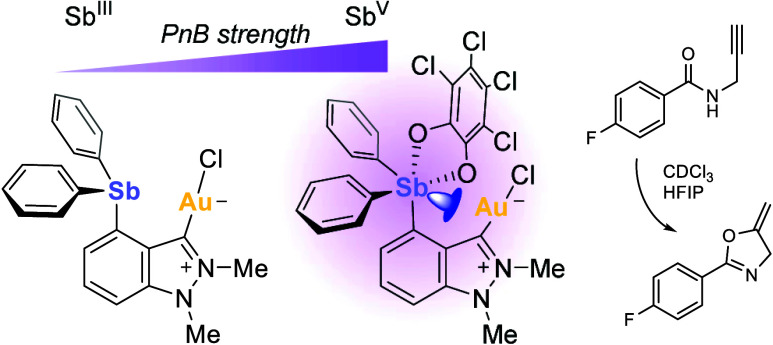

Our interest in the design of ambiphilic ligands and their coordination to gold has led us to synthesize an indazol-3-ylidene gold chloride complex functionalized at the 4-position of the indazole backbone by a stibine functionality. The antimony center of this new complex cleanly reacts with *o*-chloranil to afford the corresponding stiborane derivative. Structural analysis indicates that the stiborane coordination environment is best described as a distorted square pyramid whose open face is oriented toward the gold center, allowing for the formation of a long donor–acceptor, or pnictogen, Au → Sb bonding interaction. The presence of this interaction, which has been probed computationally, is also manifested in the enhanced catalytic activity of this complex in the cyclization of *N*-propargyl-4-fluorobenzamide.

## Introduction

Homogeneous gold catalysis has undergone a rapid expansion over the last two decades.^1^ Within this broader area of contemporary research, increasing attention has been devoted to the design of ligand systems in which gold is positioned in close proximity to an electron-deficient or Lewis acidic functionality that can potentially engage the gold center directly or through one of its ancillary ligands.^2^ Most notable successes in this chemistry include complexes supported by phosphinoborane^3^ or phosphinoalane^4^ ligands. Exploration of these systems has revealed the key role of the group 13 functionality in carbophilic catalysis by enhancement of the electrophilic character of the gold center through direct Au → B interaction^3c,5^ or ionization of Au–Cl bonds.^6^

**Figure 1:**
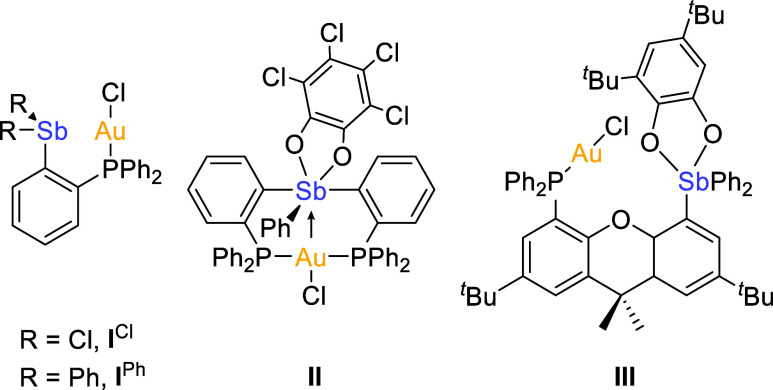
Gold complexes supported by phosphoryl-antimony scaffolds.

Our contributions to this area of research have focused mainly on investigating phosphino-antimony ligands, where the antimony center behaves as a σ-acidic or pnictogen bond (PnB) donor^7^ functionality toward gold.^8^ In some cases, this functionality can also promote the cleavage of the Au–Cl bond, leading to enhanced catalysis.^9^ Examples of such systems are depicted in Figure 1, illustrating three types of scaffolds that support such interactions.^8c,8e,9,10^ In particular, we have reported examples documenting the ability of dichlorostibines as in **I**^Cl ^(9a) or catecholatostiboranes, as in **II**(8e) and **III**,^9b^ to activate a nearby gold chloride moiety. To further elaborate on this family of compounds, we have now questioned whether *N*-heterocyclic-carbene-based systems analogous to **III** could be accessed for potential applications in catalysis. These studies were prompted by our desire to better understand the influence of the supporting scaffold on the properties of the complexes. In this paper, we describe a new complex in which a *N*-heterocyclic-carbene-bound gold chloride moiety is flanked by a pendent antimony functionality. Our results indicate moderate enhancement of carbophilic catalysis when the antimony fragment is oxidized from the +3 to the +5 state.

## Results and Discussion

### Synthesis and Analysis

**Figure 2:**
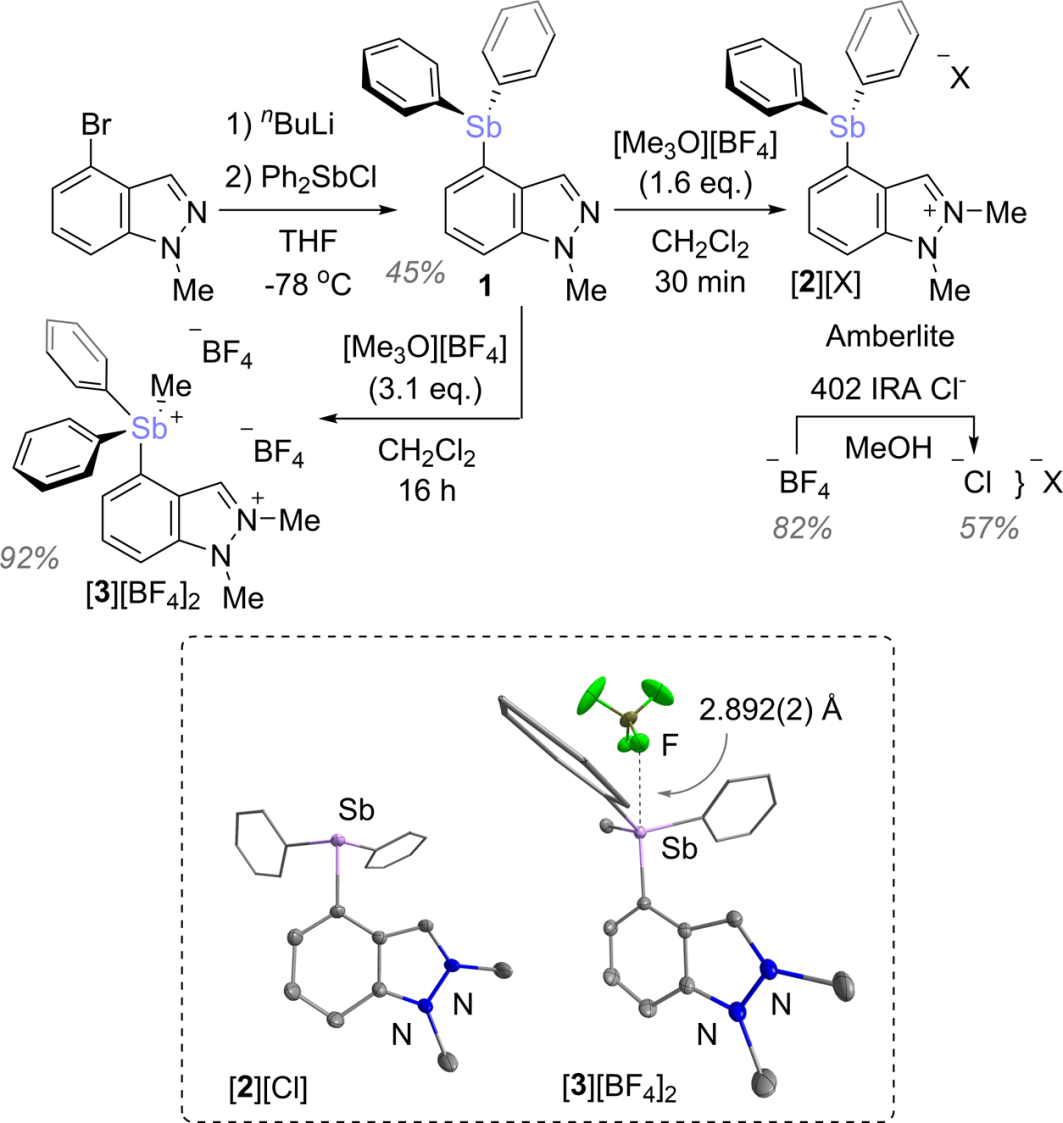
Synthesis of **1**, [**2**][X] (X = BF_4_^–^ or Cl^–^) and [**3**][BF_4_]_2_. The inset shows the crystal structures of [**2**][Cl] and [**3**][BF_4_]_2_. Ellipsoids are drawn at the 50% probability level. Solvent molecules, counterions, and H atoms are omitted for clarity.

Building on our previous efforts^11^ in the chemistry of indazole-based ligands,^12^ we decided to direct our attention toward the use of an indazol-3-ylidene backbone with the objective of benefiting from the strong sigma donor properties that tipify *N*-heterocyclic carbene ligands. The new antimony-based platform was synthesized through the lithiation of 4-bromo-1-methyl-1*H*-indazole^12a^ and subsequent addition of Ph_2_SbCl, yielding the desired product 4-diphenylstibino-1-methyl-1*H*-indazole (**1**) in 45% yield (Figure 2). The formation of the resulting stibine was confirmed by ^1^H NMR spectroscopy which indicated the presence of a diagnostic singlet for the indazole N=C*H* unit at 7.76 ppm accompanied by a distinctive methyl peak at 4.06 ppm.

Subsequent methylation of **1** using [Me_3_O][BF_4_] afforded [**2**][BF_4_] in an 82% yield under the specified conditions. Anion exchange afforded [**2**][Cl] which was fully characterized (Supporting Information). Successful methylation of the system was confirmed by the detection of two diagnostic methyl ^1^H NMR resonances at 4.15 and 4.21 ppm. The indazolium N=C*H* signal also shifts downfield to 8.63 ppm. The structure of this derivative and the successful methylation of the indazole backbone were confirmed by single-crystal X-ray diffraction (Figure 2). The methylation of **1** needs careful controlling. Indeed, addition of excess methylating agents or prolonged reaction times led to the formation of the indazolium stibonium dication [**3**]^2+^ which was isolated as a tetrafluoroborate salt and fully characterized. The formation of this dication is accompanied by the appearance of a new ^1^H NMR resonance at 2.70 ppm, consistent with an Sb-C*H*_3_ moiety.^13^ We were able to obtain single crystals of [**3**][BF_4_]_2_ which confirm the formation of an indazolium stibonium dicationic entity. The antimony atom of this dication forms a short contact of 2.892(2) Å with the fluorine atom of one of the tetrafluoroborate counterions as sometimes observed in the structure of stibonium tetrafluoroborate salts.^14^ This distance is shorter than the sum of the van der Waals radii of antimony and fluorine (3.53 Å).^15^ It is also shorter than the Sb···F_BF_4__ separation that ranges from 3.19 to 3.53 Å observed in [Ph_3_SbMe][BF_4_],^16^ suggesting that the dicationic nature of [**3**]^2+^ elevates the Lewis acidity of the antimony center leading to a stronger Sb···F_BF_4__ secondary interaction^17^ or pnictogen bond.^7^

**Figure 3:**
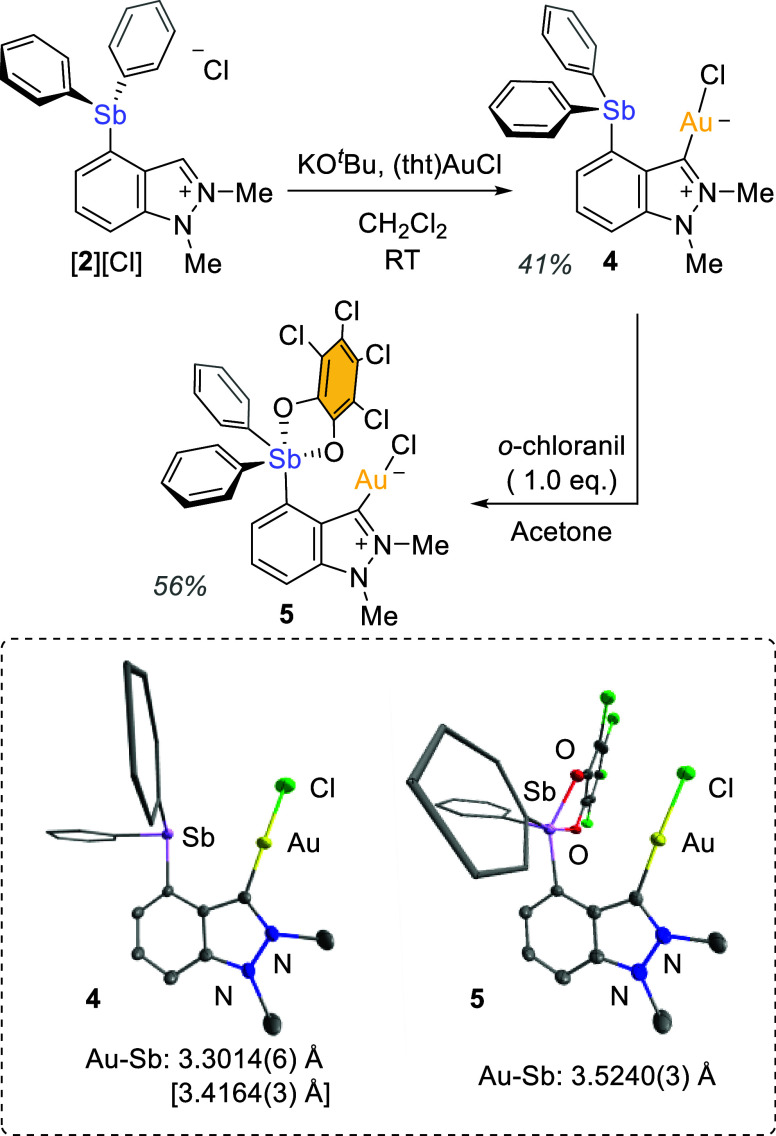
Synthesis of **4** and **5**. The crystal structures of **4** and **5** are shown in the inset. Ellipsoids are drawn at the 50% probability level. Solvent molecules and H atoms are omitted for clarity.

Stimulated by the possibility of installing an AuCl unit at the 3-position of these two platforms, we attempted auration of [**2**]^+^ and [**3**]^2+^ using protocols that have been described previously.^11a,11b,12^ Unfortunately, the indazolium stibonium dication [**3**]^2+^ did not react cleanly when treated with a base and (tht)AuCl (tht = tetrahydrothiophene). A more gratifying outcome resulted from the reaction of [**2**][Cl] with KO^*t*^Bu and (tht)AuCl. Indeed, this reaction afforded the corresponding neutral gold(I) stibine complex **4** which could be isolated in moderate yields (Figure 3). The new gold complex **4** was fully characterized. The ^1^H NMR spectrum shows the disappearance of the characteristic downfield singlet associated with the indazolium N=C*H* moiety. A substantial downfield shift is also observed for the carbene center which shifts from 134.63 ppm in [**2**][Cl] to 169.46 ppm in **4**, a ^13^C chemical shift similar to that observed shift for the gold-bound carbon atom of other indazole-ylidene gold complexes.^12a^ The solid-state structure of **4** exhibits two independent molecules in the asymmetric unit referred to as molecule I and molecule II that assume very similar structures. The Au–Sb distances in the two independent molecules (3.3014(6) and 3.4164(3) Å for molecules I and II, respectively) are significantly longer than coordination bonds in stibine gold complexes such as [Au(SbPh_3_)_4_][ClO_4_] (2.656–2.658 Å).^18^ They are also longer than those found in the structure of **I**^Ph^ which displayed nonbonded Au–Sb distances of 3.053(1) and 3.049(1) Å.^9a^ It remains that the distances separating the gold and antimony atoms of **4** are within the van der Waals radii of the two elements (3.72 Å),^15,19^ suggesting the possibility of a direct interaction.

We next focused on the oxidation of the complex using *o*-chloranil which selectively reacted with the stibine moiety, converting **4** into a tetrachlorocatecholatostiborane gold complex (**5**). It is worth pointing out that *o*-chloranil reacts selectively with the antimony center^8e,9b,10^ leaving the gold untouched.^20^ Formation of **5** led to a downfield shift of the aromatic peaks in the ^1^H NMR spectrum. Furthermore, the ^13^C{^1^H} NMR spectrum shows a resonance at 166.06 ppm, supporting the existence of an intact carbene gold chloride moiety. In the crystal, the stiborane assumes a distorted square pyramidal geometry with a τ_5_ value of 0.27. The orientation of this unit is such that the most open face of the antimony-centered distorted square pyramid is oriented toward the gold atom. The distance between the antimony and gold atoms is 3.5240(3) Å, which is significantly longer than that found in **II** (2.8608(5) Å) in which the two elements are intimately connected via a strong Au → Sb interaction.^8e^ The Au–Sb separation is also slightly elongated compared to that of **4**, presumably as a result of the increased steric bulk around the antimony center. This elongated distance remains just inside the sum of the van der Waals radii of the two elements (3.72 Å)^15,19^ suggesting that a direct, albeit weak, interaction between the two heavy elements may still exist.

### Computational Investigation

**Figure 4:**
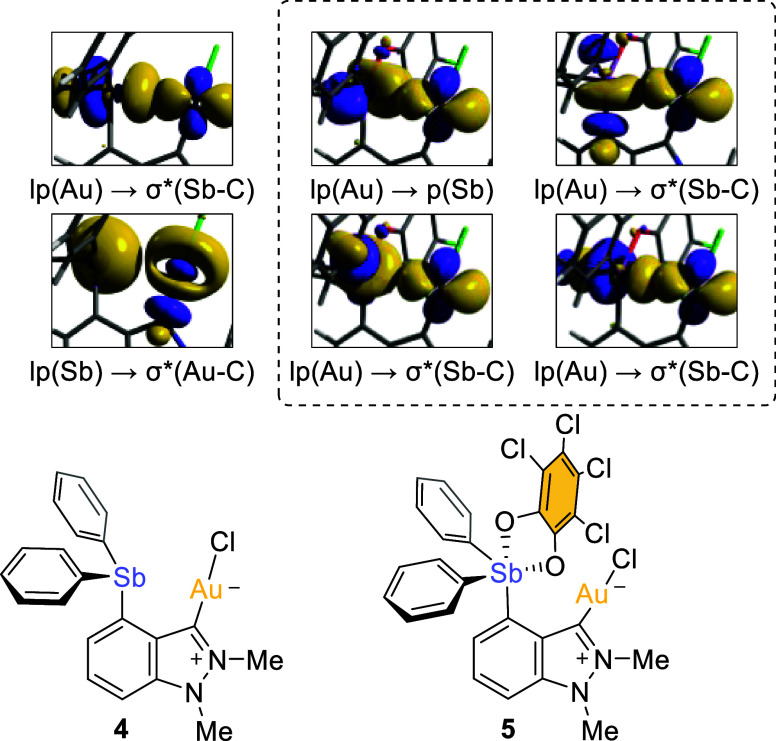
NBOs involved in second order Au-Sb interactions in **4** and **5**. The contour plots are draw at an isovalue of 0.005.

To understand the nature of the interaction occurring between the gold and antimony centers, we computed the structures of **4** and **5** using density functional theory (DFT) methods (functional: MPW1PW91, basis set: cc-pVTZ-PP for gold and antimony, 6-31G(d,p) for chloride, 6-31+G(d′) for nitrogen, and 6-31G(d′) for all other atoms). Analytical frequency analyses, which yielded only positive values, were used to verify that these structures are minima. Furthermore, the computed structures closely match those determined by single-crystal X-ray diffraction, suggesting the adequacy of the chosen functional and basis sets. Second-order perturbation theory, as implemented in the natural bond orbital (NBO) method, revealed differences in the Au–Sb interaction in **4** and **5** (Figure 4). Two main interactions were seen in **4**: one showing donation from a gold lone pair to an Sb–C σ* orbital with an energy of 4.39 kcal mol^–1^ and the other showing donation from an antimony lone pair to a Au–C σ* orbital with an energy of 3.67 kcal mol^–1^ (Figure 4). This analysis highlights the ambiphilic character of the trivalent antimony center, which behaves both as a Lewis acid and base. In contrast, the pentavalent antimony center of **5** exhibits only Lewis acidic character as supported by the presence of four Au → Sb donor acceptor interactions (Figure 4). A deletion energy calculation suggest that these interactions add 7.46 kcal mol^-1^ to the stability of the complex.

**Figure 5:**
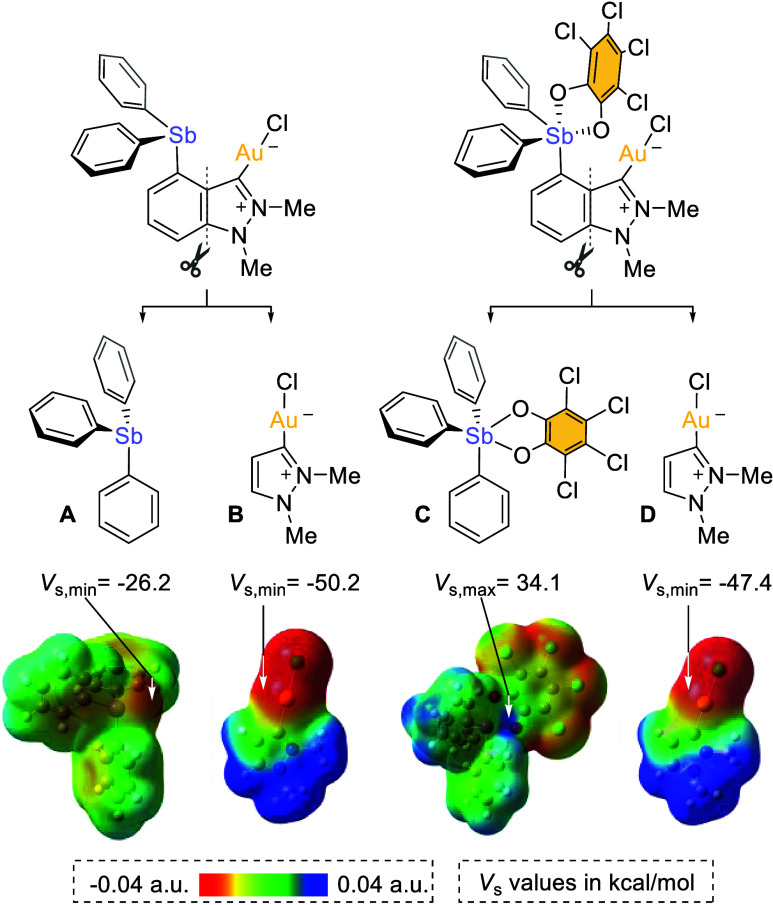
ESP maps of model structures **A**, **B**, **C**, and **D**. All ESP maps are drawn with an isosurface value of 0.001.

To further support the increase in Lewis acidity of the antimony center upon oxidation of **4** into **5**, we turned our attention to electrostatic potential (ESP) maps, projecting that they would illustrate the electrostatic component of the Au–Sb interaction. Inspection of the ESP maps of **4** and **5** showed no observable areas of charge concentration on the heavy atoms because of their proximity and mutual neutralization (Supporting Information). Faced with this difficulty, we decided to computationally split these compounds into two separate units along the C–C bond connecting the benzene and pyrazole ring of the indazole backbone (Figure 5). Each of the resulting fragments (**A**, **B**, **C**, and **D**) were capped with hydrogen atoms at the disconnection sites but were otherwise kept at the geometries they assumed in the optimized structures of the parent complexes. The molecular electrostatic potential of **C** allows for the clear identification of a σ-hole associated with a *V*_s,max_ of 34.1 kcal mol^–1^ at the pentavalent antimony center. By contrast, there is no indication of the presence of an electropositive region around the trivalent antimony atom in model **A**; instead, the surface is negatively charged as indicated by a *V*_s,min_ value of −26.2 kcal mol^–1^ which we associate with the antimony lone pair. Model **B** and **D**, on the other hand, display an electronegative region around the gold center with a *V*_s,min_ value of −50.2 kcal mol^–1^ for **B** and *V*_s,min_ of −47.4 kcal mol^–1^ for **D**. Based on this analysis, we propose that in **5**, a favorable σ-hole interaction occurs between the antimony and the gold centers since the surface potentials of their respective fragments **C** and **D** are large and opposite in sign. Altogether the computational results obtained in this investigation indicate the presence of a Au → Sb interaction in **5** of both charge transfer and electrostatic origin. This interaction can be described as an atypical pnictogen bond (PnB) in which the transition metal acts as the PnB acceptor and antimony as the PnB donor.^7b^ Such interactions which were first characterized by us in derivatives of type **I** and **II**,^8a,8c,8e,9a,21^ have recently attracted renewed attention.^22^

### Catalysis

**Figure 6:**
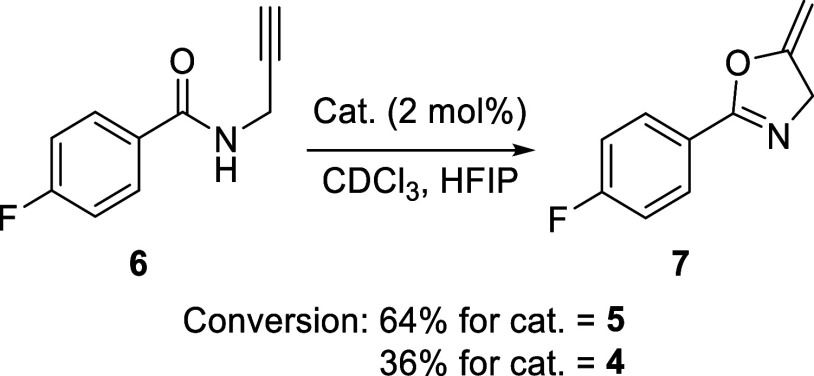
Cyclisation of *N*-propargyl-4-fluorobenzamide catalyzed by **4** and **5**.

To test if the above-discussed Au–Sb pnictogen bond could influence the reactivity of the metal center, we tested **4** and **5** as catalysts for the cyclization of *N*-propargyl-4-fluorobenzamide (**6**), a well-studied reaction that has emerged as a convenient benchmark in carbophilic catalysis (Figure 6).^23^ Due to its relative simplicity, we were able to track its progress through ^1^H NMR spectroscopy. The first experiments were carried out at room temperature in CDCl_3_ with a catalyst loading of 2 mol %. Under these conditions, the formation of the desired methyleneoxazole **7** was slow. Integration of the ^1^H NMR spectra after 3.5 h afforded conversions of 5 and 13% for **4** and **5**, respectively. Encouraged by these initial results which suggested a positive correlation of the catalytic properties with the oxidation state of the antimony center, we decided to test if the superiority of **5** would persist in the presence of hexafluoroisopronanol (HFIP), a solvent previously used to activate the Au–Cl bond.^24^ Toward this end, we investigated the cycloisomerization of *N*-propargyl-4-fluorobenzamide in 11:1 CDCl_3_/HFIP (v/v), again using a catalyst loading of 2 mol %. Under these conditions, the reaction was substantially faster, as it afforded conversions of 36% and 64% for **4** and **5**, respectively, after 3.5 h. While these results validate the positive attribute of HFIP as an activator, they also show that **5** remains a better catalyst or precatalyst under these conditions. These results support our hypothesis that carbophilic reactivity of the catalyst is increased when the gold center is in close proximity to the more Lewis-acidic stiborane.

## Conclusions

In summary, we have developed a new gold carbene complex in which the gold atom is flanked by an adjacent stibine. The presence of this inherently redox active pnictogen-containing moiety provides an easy way to toggle the electronic features and properties of the platform by oxidation of the stibine into a stiborane using *o*-chloranil. When in the pentavalent state, the antimony atom engages the gold center in a long Au → Sb interaction, or pnictogen bond, as supported both structurally and computationally. We contend that the formation of this interaction elicits an enhancement in the carbophilic reactivity of the gold center readily reflected in the greater catalytic activity of the stiborane-gold derivative **5** in the cyclization of propargyl amide **6**. Alternatively, we also consider the possible involvement of the pentavalent antimony center of **5** in the Lewis acid activation of the Au–Cl bond. Irrespective of the *modus operandi*, these results illustrate how peripheral changes beyond the active site and weak interactions can be used to adjust the catalysis. Finally, we note that, although ambiphilic ligands containing antimony have been known for some time, their chemstry is attracting a renewed interest.^39^

## Experimental Section

### General Considerations

4-Bromo-1-methyl-1*H*-indazole^12a^ and (tht)AuCl (tht = tetrahydrothiophene)^25^ were prepared following literature methods. Trimethyloxonium tetrafluoroborate, potassium *tert*-butoxide, and *o*-chloranil were purchased from Thermo Scientific Chemicals and BeanTown Chemical and used as received. Solvents were dried over Na/K (THF) or CaH_2_ (CH_2_Cl_2_) by refluxing under N_2_. All other solvents were ACS reagent grade and were used as received. All compounds were synthesized under a dry N_2_ atmosphere with standard Schlenk techniques or in a nitrogen-filled glovebox. ^1^H and ^13^C NMR spectra were recorded at room temperature on a Bruker Avance 500 NMR spectrometer or a Bruker Ascend 400 NMR spectrometer. Chemical shifts are given in parts per million and are referenced to residual ^1^H and ^13^C solvent signals. Mass spectrometric analyses were performed in-house at the Center for Mass Spectrometry. Elemental analyses were performed at Atlantic Microlab (Norcross, GA).

### Crystallographic Measurements

Crystallographic measurements were performed at 110 K using a Bruker D8 QUEST diffractometer (Mo Kα radiation, λ = 0.71069 Å) equipped with Photon III detectors and a Rigaku XtaLAB Synergy-S diffractometer (Ag-Kα radiation, λ = 0.56087) with a Dectris Eiger 2 detector. A specimen of suitable size and quality was selected and mounted onto a nylon loop in each case. Integrated intensity information for each reflection was obtained by reducing the data frames with the program Bruker AXS APEX3^26^ and CrysAlis^Pro^.^27^ The semiempirical method SADABS was used for absorption corrections.^28^ The structures were solved by direct methods (ShelXT)^29^ and refined by the full-matrix least-squares technique against *F*^2^ with anisotropic temperature-dependent parameters for all non-hydrogen atoms (ShelXL)^30^ using the Olex2 interface.^31^ All H atoms were geometrically placed and refined using the riding atom model. Diamond 4 was employed for the final data presentation and structure plots. The data have been deposited with the Cambridge Structural Database. CCDC 2376381–2376384 contain the supplementary crystallographic data for this paper.

### Computational Methods

Computations were performed using density functional theory (DFT) in the Gaussian 16 program.^32^ All calculations were conducted with the MPW1PW91 functional and a mixed basis set (cc-pVTZ-PP with a set of f-orbital polarization functions for Au^33^ and Sb,^34^ 6-31G(d,p) for chloride, 6-31+G(d′) for nitrogen and 6-31G(d′) for all other atoms) starting from the crystal structure geometries when available. No imaginary frequencies were found for the optimized structures, confirming that a local minimum on the potential energy hypersurface had, in all cases, been reached. The optimized structures were subjected to NBO analysis using NBO 7^35^ and visualized and plotted using the Avogadro program.^36^ Electrostatic potential (ESP) maps were generated based on the gas-phase optimized geometries of the structures of **4** and **5** and modified to afford the desired models **A**, **B**, **C**, and **D** which were capped by hydrogen atoms at the open sites using the valence completion function of GaussView.^37^ The resulting structures were subjected to a single-point calculation. The ESP maps of **4**, **5**, **A**, **B**, **C**, and **D** were determined at an isodensity value of 0.001 electrons/bohr^3^. ESP maps were generated and visualized in GaussView.^37^ Multiwfn^38^ was used to identify any existing areas of maximum or minimum electrostatic potential (*V*_S,max_, *V*_S,min_).

#### Synthesis of **1**

^*n*^BuLi (3.2 mL, 8.0 mmol, 2.5 M in hexanes) was added to a solution of 4-bromo-1-methyl-1*H*-indazole (1.5 g, 7.1 mmol) in THF (20 mL) at −78 °C leading to a color change from pale yellow to bright orange. The resulting solution was stirred at that temperature for 1 h, at which point a solution of Ph_2_SbCl (2.2 g, 7.1 mmol) in THF (10 mL) was added by cannula transfer. The reaction mixture was warmed to room temperature and stirred overnight, and then quenched by adding a saturated aqueous ammonium chloride solution (10 mL). After removal of the THF via rotary evaporation, the remaining aqueous phase was extracted with CH_2_Cl_2_ (3 × 30 mL). The organic fractions were combined, dried over MgSO_4_, and dried *in vacuo* to give a pale-yellow oil, which was further purified by column chromatography in air (silica 230–400 mesh, 4:1 hexane/ethyl acetate). This procedure afforded **1** as a pale-yellow solid (1.35 g, 45%). ^1^H NMR (499.41 MHz, CDCl_3_) δ 7.76 (s, 1H, indazole −N=C*H*), 7.48–7.46 (m, 4H, phenyl), 7.39 (d, *J* = 8.5 Hz, 1H, indazole-*H*), 7.34–7.28 (m, 7H, phenyl and indazole-*H*), 7.10 (dd, *J* = 6.8, 0.7 Hz, 1H, indazole-*H*), 4.06 (s, 3H, N–C*H*_3_). ^13^C{^1^H} NMR (125.76 MHz, CDCl_3_) δ 139.27 (s, *N*-*ipso*-indazole-*C*), 137.40 (s, *ipso*-phenyl-*C*), 136.55 (s, *ortho*-phenyl-*C*), 134.14 (s, indazole-N=*C*H), 132.03 (s, *ipso*-indazole-*C*), 129.66 (s, *ipso*-Sb-*C*), 129.18 (s, indazole-*C*), 129.09, (s, *meta*-phenyl-*C*), 128.91(s, *para*-phenyl-*C*), 126.50 (s, indazole-*C*), 109.54 (s, indazole-*C*), 35.74 (s, N-*C*H_3_). Electrospray ionization mass spectrometry (ESI-MS) calcd for C_20_H_17_N_2_Sb [M + H]^+^ 407.0503, found: 407.0498.

#### Synthesis of **[2][Cl]**

Step 1: Trimethyloxonium tetrafluoroborate (0.28 g, 1.9 mmol) was added to a solution of **1** (0.50 g, 1.2 mmol) in CH_2_Cl_2_ (10 mL). The mixture was stirred for 30 min at room temperature and then quenched with methanol (5 mL). The solvent was removed *in vacuo*, and the resulting solid was dissolved in CH_2_Cl_2_ and filtered through Celite. Tituration with Et_2_O followed by filtration and drying afforded crude [**2**][BF_4_] as a white solid (0.51 g, 82%). The product was analyzed by ^1^H NMR spectroscopy in CD_3_CN and used in the next step without further purification. ^1^H NMR (499.41 MHz, CD_3_CN) δ 8.48 (s, 1H, indazolium-N=C*H*), 7.73 (m, 2H, indazolium-*H*), 7.48 (m, 4H, phenyl), 7.40 (m, 7H, phenyl and indazolium-*H*), 4.17 (s, 3H, N^+^–C*H*_3_), 4.13 (s, 3H, N–C*H*_3_). Step 2: Amberlite 402 IRA Cl^–^ (8.0 g) was washed with methanol (3 × 10 mL) and added to a solution of [**2**][BF_4_] in methanol (0.51 g in 15 mL). The reaction mixture was stirred at room temperature overnight. The solution was filtered through a glass frit to remove the ion-exchange resin. The filtrate was brought to dryness to afford a yellow, oily residue that solidified upon addition of acetone (20 mL). The product, [**2**][Cl], was recovered by filtration as a white solid (0.26 g, 57% yield). Single crystals suitable for X-ray analysis were obtained by the slow diffusion of pentane into a CH_2_Cl_2_ solution of [**2**][Cl]. ^1^H NMR (499.41 MHz, CD_3_CN). δ 8.63 (s, 1H, indazolium-N=C*H*), 7.74 (m, 2H, indazolium-*H*), 7.48 (m, 4H, phenyl), 7.40 (m, 7H, phenyl and indazolium-*H*), 4.21 (s, 3H, N^+^–C*H*_3_), 4.15 (s, 3H, N–C*H*_3_). ^13^C{^1^H} NMR (125.76 MHz, CD_3_CN) δ 141.43 (s, *N*-*ipso*-indazolium-*C*), 137.65 (s, *ipso*-phenyl-*C*), 137.29 (s, *ortho*-phenyl-*C*), 136.04 (s, *ipso*-indazolium-*C*), 134.63 (s, indazolium-N=*C*H), 134.60 (s, indazolium-*C*), 133.97 (s, indazolium-*C*), 130.33 (s, *para*-phenyl-*C*), 130.28 (s, *meta*-phenyl-*C*), 125.48 (s, *ipso*-Sb-*C*), 112.17 (s, indazolium-*C*), 38.93 (s, N^+^-*C*H_3_), 34.34 (s, N-*C*H_3_). ESI-MS calcd for C_21_H_20_N_2_Sb^+^ [M]^+^ 421.0659, found: 421.0657.

#### Synthesis of **[3][BF**_**4**_**]**_**2**_

Trimethyloxonium tetrafluoroborate (0.74 g, 5.0 mmol) was added to a solution of **1** (0.64 g, 1.6 mmol) in CH_2_Cl_2_ (10 mL). The mixture was stirred for 16 h at room temperature and then quenched with methanol (5 mL). The resulting mixture was brought to dryness by the evaporation of the solvents. The residue was dissolved in acetonitrile, filtered through Celite, and evacuated to dryness to afford [**3**][BF_4_]_2_ as a white solid (0.63 g, 92% yield). Single crystals suitable for X-ray analysis were obtained by the slow diffusion of pentane into a CH_2_Cl_2_ solution of [**3**][BF_4_]_2_. ^1^H NMR (499.41 MHz, CD_3_CN). δ = 8.46 (s, 1H, indazolium-N=C*H*), 8.09 (d, *J* = 8.9 Hz, 1H, indazole-*H*), 7.98 (t, *J* = 15.9 Hz, 1H, indazole-*H*), 7.80 (d, *J* = 7.0 Hz, 1H, indazole-*H*), 7.76 (m, 6H, phenyl), 7.67 (m, 4H, phenyl), 4.26 (s, 3H, N^+^–C*H*_3_), 4.24 (s, 3H, N–C*H*_3_), 2.70 (s, 3H, Sb*-*C*H*_3_). ^13^C{^1^H} NMR (125.76 MHz, CD_3_CN) δ 141.77 (s, *N*-*ipso*-indazolium-*C*), 136.53 (s, indazolium-*C*), 136.34 (s, *meta*-phenyl-*C*), 134.58 (s, *para*-phenyl-*C*), 133.68 (s, indazolium-*C*), 132.91 (s, indazolium-N=*C*H), 131.71 (s, *ortho*-phenyl-*C*), 123.96 (s, *ipso*-phenyl-*C*), 122.26 (s, *ipso*-indazolium-*C*), 119.79 (s, *ipso*-Sb-*C*), 116.94 (s, indazolium-*C*), 39.30 (s, N^+^-*C*H_3_), 34.72 (s, N-*C*H_3_), 4.54 (s, Sb-*C*H_3_). ESI-MS calcd for C_22_H_23_N_2_Sb^2+^ [M]^2+^ 218.0444, found: 218.0443.

#### Synthesis of **4**

In a glovebox, [**2**][Cl] (66 mg, 0.14 mmol), potassium *tert*-butoxide (33 mg, 0.29 mmol), and (tht)AuCl (70 mg, 0.22 mmol) were dissolved in dry THF (5.0 mL). The resulting mixture was stirred at room temperature for 1 h. The resulting solution was filtered in air over a pad of silica (top) and Celite (bottom) to remove a black precipitate. The filtrate was dried *in vacuo* and purified by recrystallization from CH_2_Cl_2_/Et_2_O (on the benchtop) to yield the corresponding complex **4** as a pale-yellow powder (39 mg, 41% yield). Single crystals suitable for X-ray analysis were obtained by the slow diffusion of pentane into a CH_2_Cl_2_ solution of **4**. ^1^H NMR (499.41 MHz, CD_3_CN) δ 7.53 (m, 2H, indazolium-*H*), 7.44 (m, 4H, phenyl), 7.33 (m, 6H, phenyl), 7.00 (dd, *J* = 6.2, 1.3 Hz, 1H, indazolium-*H*) 4.27 (s, 3H, N^+^–C*H*_3_), 4.02 (s, 3H, N–C*H*_3_). ^13^C{^1^H} NMR (125.76 MHz CD_3_CN) δ 169.46 (s, Au-*C*), 141.16 (s, *ipso*-phenyl-*C*), 140.03 (s, *N*-*ipso*-indazolium-*C*), 137.71 (s, *ortho*-phenyl-*C*), 134.74 (s, *ipso*-indazolium-*C*), 134.22 (s, *ipso*-Sb-*C*), 130.80 (s, indazolium-*C*), 129.83 (s, *para*-phenyl-*C*), 129.64 (s, *meta*-phenyl-*C*), 111.01 (s, indazolium-*C*), 41.32 (s, N^+^-*C*H_3_), 34.46 (s, N-*C*H_3_). ESI-MS calcd for C_42_H_38_Au_2_N_4_Sb_2_^2+^ [M]^2+^ 617.0246, found: 617.0238. Elemental analysis for C_43_H_40_Au_2_Cl_4_N_4_Sb_2_: calcd: C, 37.10; H, 2.90; N, 4.02, found: C, 37.26; H, 2.83; N, 4.68.

#### Synthesis of **5**

A solution of *o*-chloranil (14 mg, 0.057 mmol) in acetone (2 mL) was added to a solution of **4** (37 mg, 0.057 mmol) in acetone (5 mL). The resulting solution was stirred at room temperature for 30 min. The solvent was removed under vacuum, and the crude product was washed with Et_2_O to afford **5** as a bright yellow/orange solid that was dried under a vacuum (28 mg, 56% yield). Single crystals suitable for X-ray analysis were obtained by the slow diffusion of hexanes into a CH_2_Cl_2_ solution of **5**. ^1^H NMR (499.41 MHz, (CD_3_)_2_SO) δ 7.91 (d, *J* = 8.2 Hz, 1H, indazolium-*H*), 7.78 (t, *J* = 14.2 Hz, 1H, indazolium-*H*) 7.61 (m, 5H, phenyl and indazolium-*H*), 7.34 (m, 6H, phenyl), 4.15 (s, 3H, N^+^–C*H*_3_), 4.10 (s, 3H, N–C*H*_3_). ^13^C{^1^H} NMR (125.76 MHz (CD_3_)_2_SO) δ 166.06 (s, Au-*C*), 145.82 (s, *o-*cloranil-*C*), 140.16 (s, *N*-*ipso*-indazolium-*C*), 139.49 (s, *ipso*-indazolium-*C*), 134.43 (s, *ortho*-phenyl-*C*), 130.88 (s, indazolium-*C*), 130.61(s, indazolium-*C*), 130.47 (s, *ipso*-Sb-*C*), 128.99 (s, 2C, *meta*-phenyl-*C* and *para*-phenyl-*C*), 128.37 (s, *ipso*-phenyl-*C*), 117.88 (s, *o-*cloranil-*C*), 115.49 (s, *o-*cloranil-*C*), 112.71 (s, indazolium-*C*), 40.69 (s, *N*^+^–C*H*_3_), 33.67 (s, *N*–C*H*_3_). ESI-MS calcd for C_27_H_20_AuCl_5_N_2_O_2_Sb [M + H]^+^ 896.8666, found 896.8654. Elemental analysis for C_27_H_19_AuCl_5_N_2_O_2_Sb: calcd: C, 36.06; N, 3.11; H, 2.13; Found: C, 35.78; N, 3.14; H, 2.25.

### Catalysis

Compounds **4** (2.3 mg, 0.0036 mmol) and **5** (3.2 mg, 0.0036 mmol) were added to two separate NMR tubes in the glovebox. To each was added 0.64 mL (0.175 mmol) of a stock solution of *N-*propalgyl-4-fluorobenzamide in CDCl_3_ (0.273 M) along with 60.0 μL of HFIP. A set of experiments were also carried out using compounds **4** and **5** as the catalysts without the addition of HFIP. The reaction progress was monitored *in situ* via ^1^H NMR. Spectra were recorded every 40 min. The final conversion of the reaction was measured based on the integration of the peaks at 7.95, 7.72, and 7.15 ppm. The spectra for each reaction are given in Figures S22–S25.

## Supporting Information Available

NMR spectra for synthesized compounds, NMR spectra for catalytic activity, and computational details (PDF)
Optimized structures in *xyz* format (XYZ)

## Supplementary Material

om4c00347_si_001.pdf

om4c00347_si_002.xyz
