# Supplementary material for: Pnictogen Bonding at the Core of a Carbene-Stiborane-Gold Complex: Impact on Structure and Reactivity
Source: Organometallics. 2024 Sep 26;43(19):2334–41. doi: 10.1021/acs.organomet.4c00347 (PMC11481098; doi:10.1021/acs.organomet.4c00347)
Supplement: Supplementary file 1 — om4c00347_si_001.pdf [file om4c00347_si_001.pdf]

Supporting Information for

Pnictogen bonding at the core of a carbene-stiborane gold complex: Impact on structure and reactivity

Paula Castro Castro,[a] and François P. Gabbaï*,[a]

[a]*Department of Chemistry, Texas A&M University, College Station, Texas 77843, United States*
**Email: francois@tamu.edu*

TABLE OF CONTENTS

NMR Spectra for Synthesized Compounds S2
NMR spectra for compound **1** S2
NMR spectra for compound [**2**][BF_4] and [**2**][Cl] S4
NMR spectra for compound [**3**][BF_4]$_2$ S7
NMR spectra for compound **4** S9
NMR spectra for compound **5** S11
NMR Spectra for Catalytic Studies S13
Computational details S15
Natural Bond Orbital (NBO) Analysis S15
Electrostatic potential maps S17

NMR Spectra for Synthesized Compounds

NMR spectra for compound 1

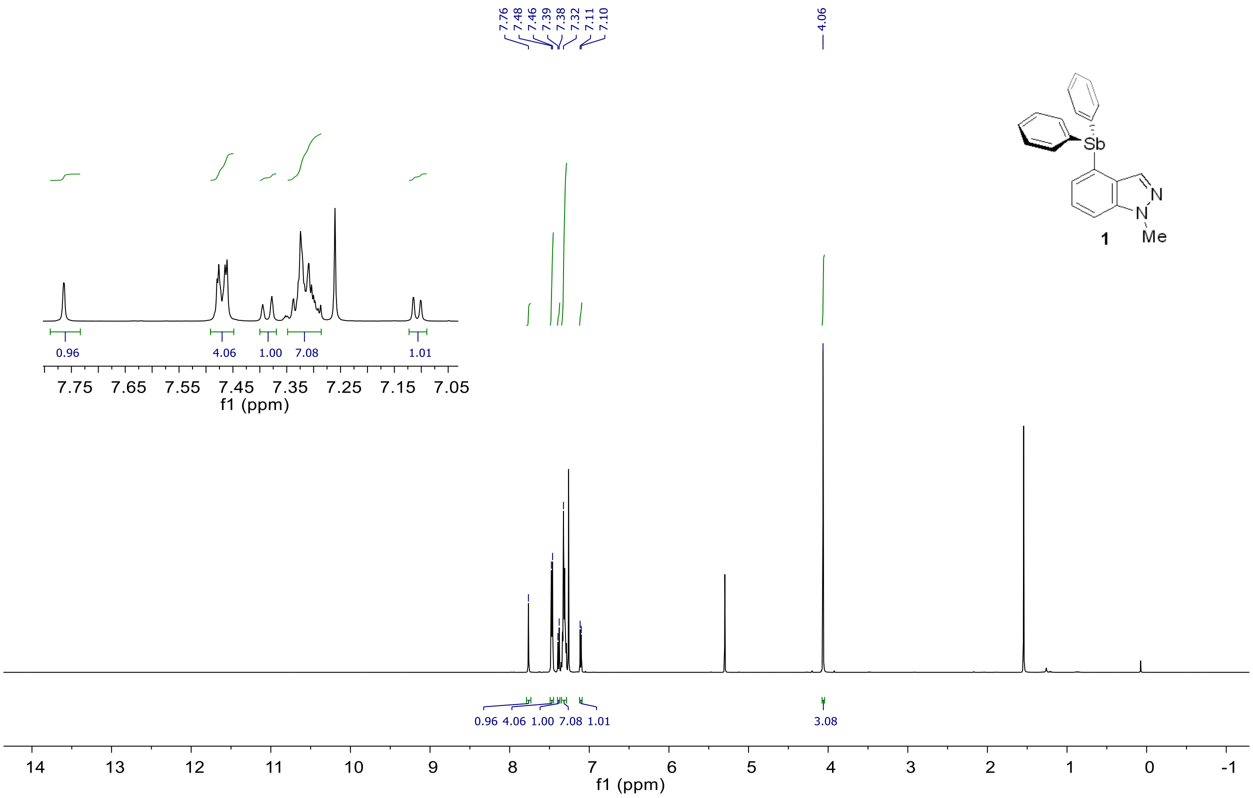

Figure S1. ^{1}H NMR spectrum of **1** in $CDCl_3$.

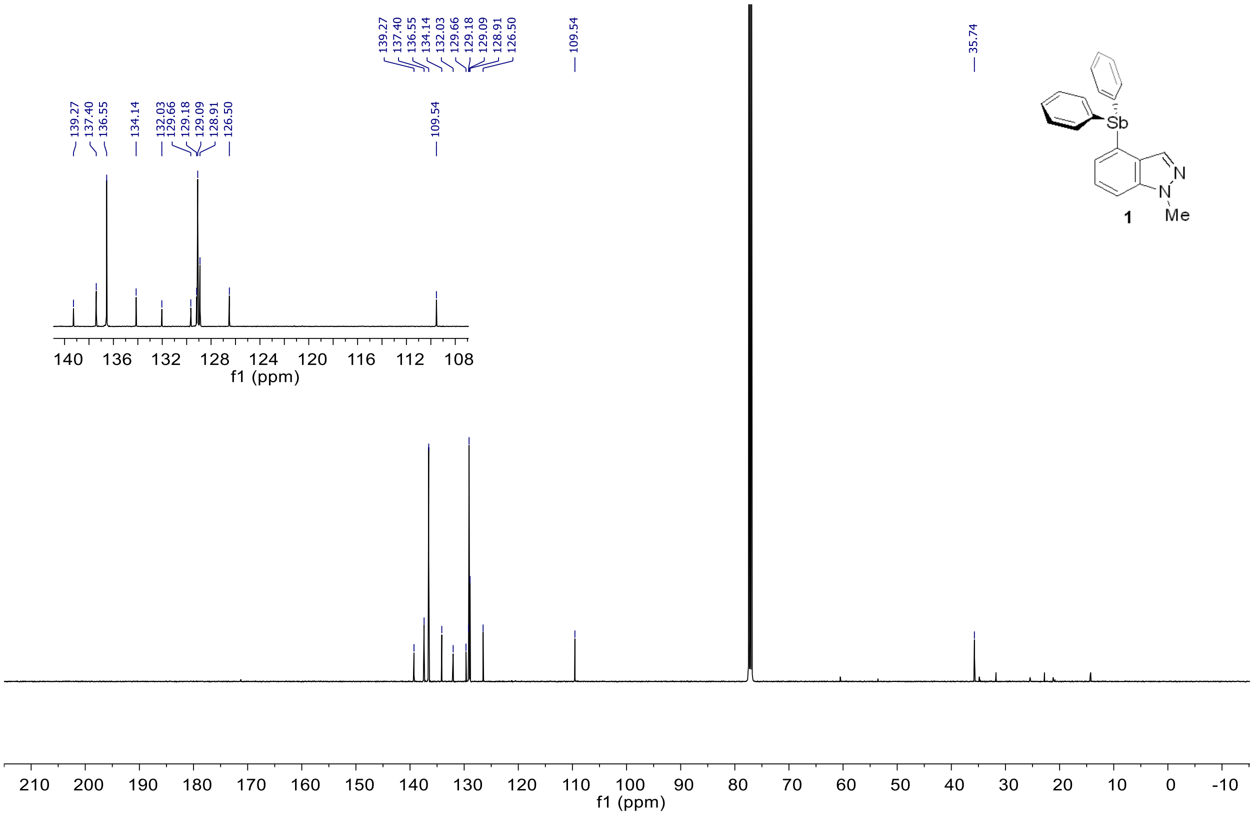

Figure S2. $^{13}C\{^1H\}$ NMR spectrum of **1** in $CDCl_3$. The solvent peak is truncated.

NMR spectra for compound [2][BF4] and [2][Cl]

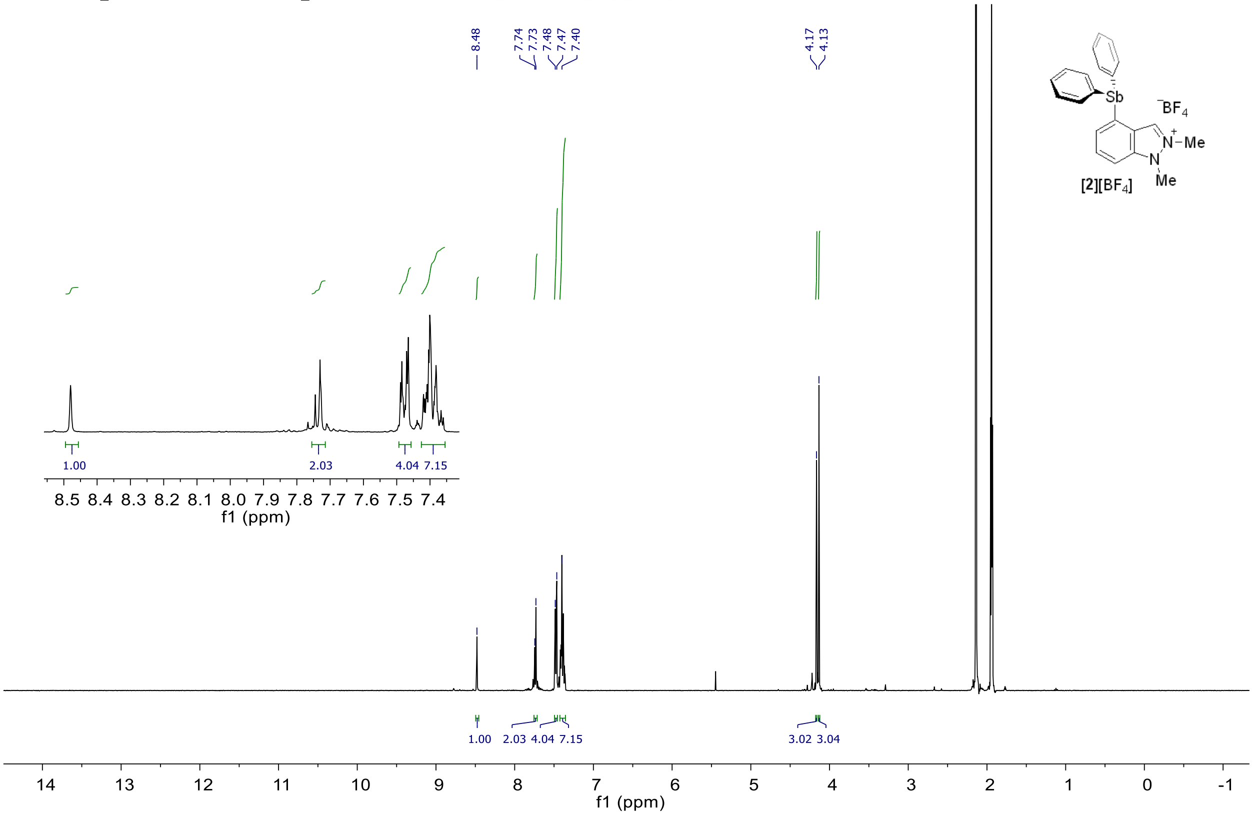

Figure S3. ^{1}H NMR spectrum of [**2**][BF_4] in CD_3CN. The solvent peak is truncated.

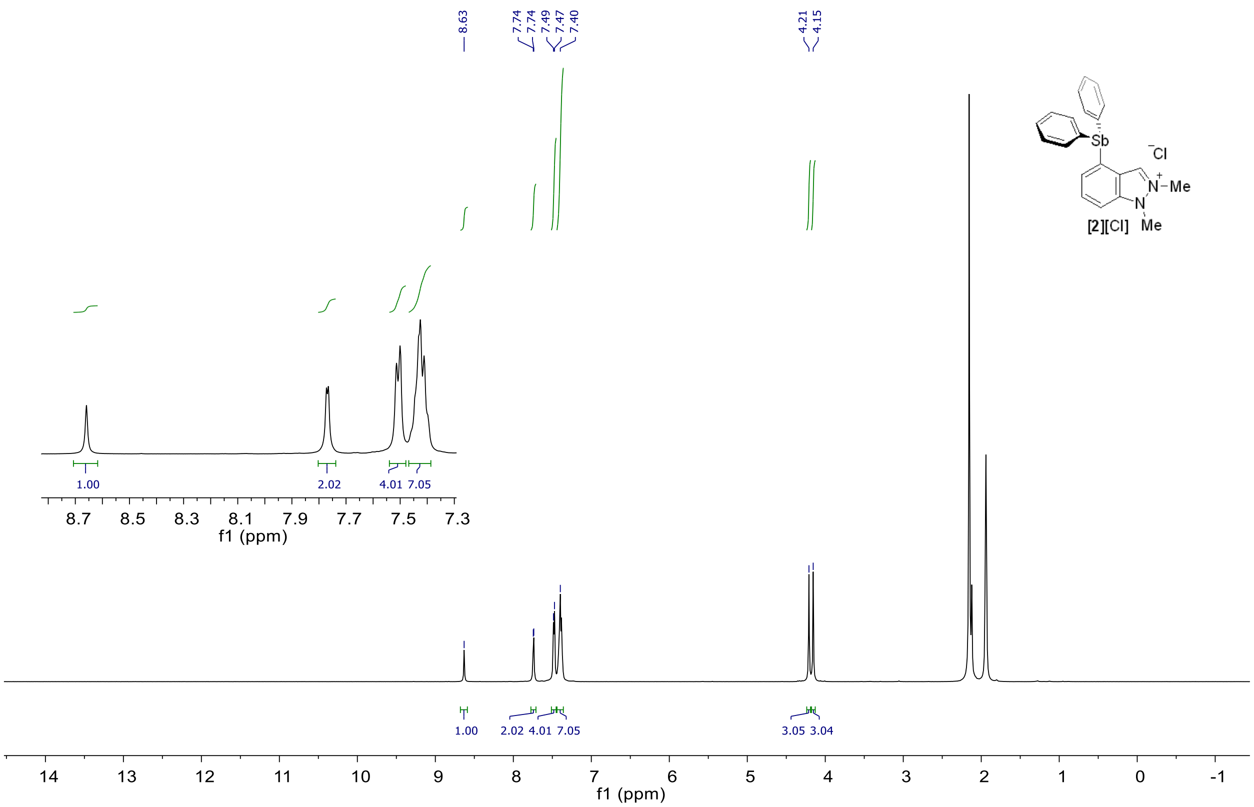

Figure S4. ^{1}H NMR spectrum of [**2**][Cl] in CD_3CN.

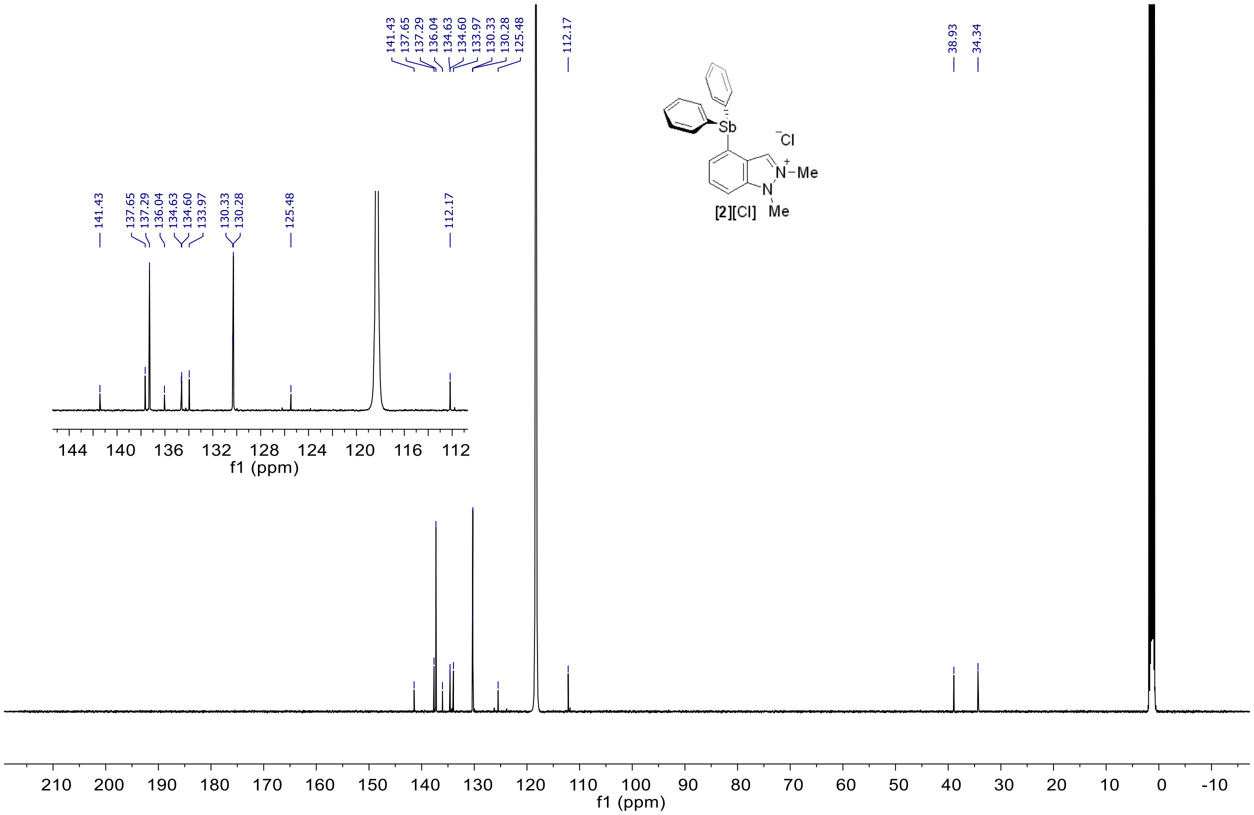

Figure S5. $^{13}C\{^{1}H\}$ NMR spectrum of [**2**][Cl] in CD_3CN. The solvent peak is truncated.

NMR spectra for compound [3][BF_4]$_2$

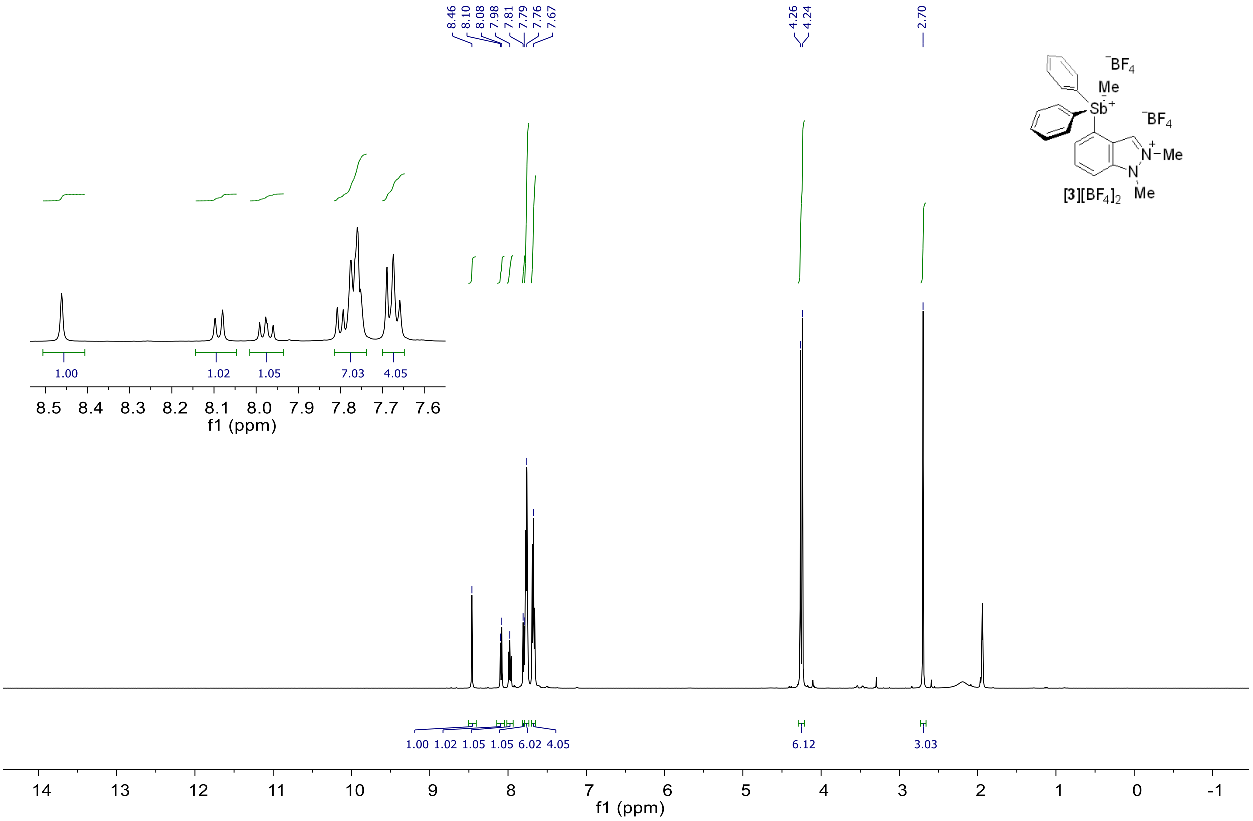

Figure S6. ^{1}H NMR spectrum of [**3**][BF_4]$_2$ in CD_3CN.

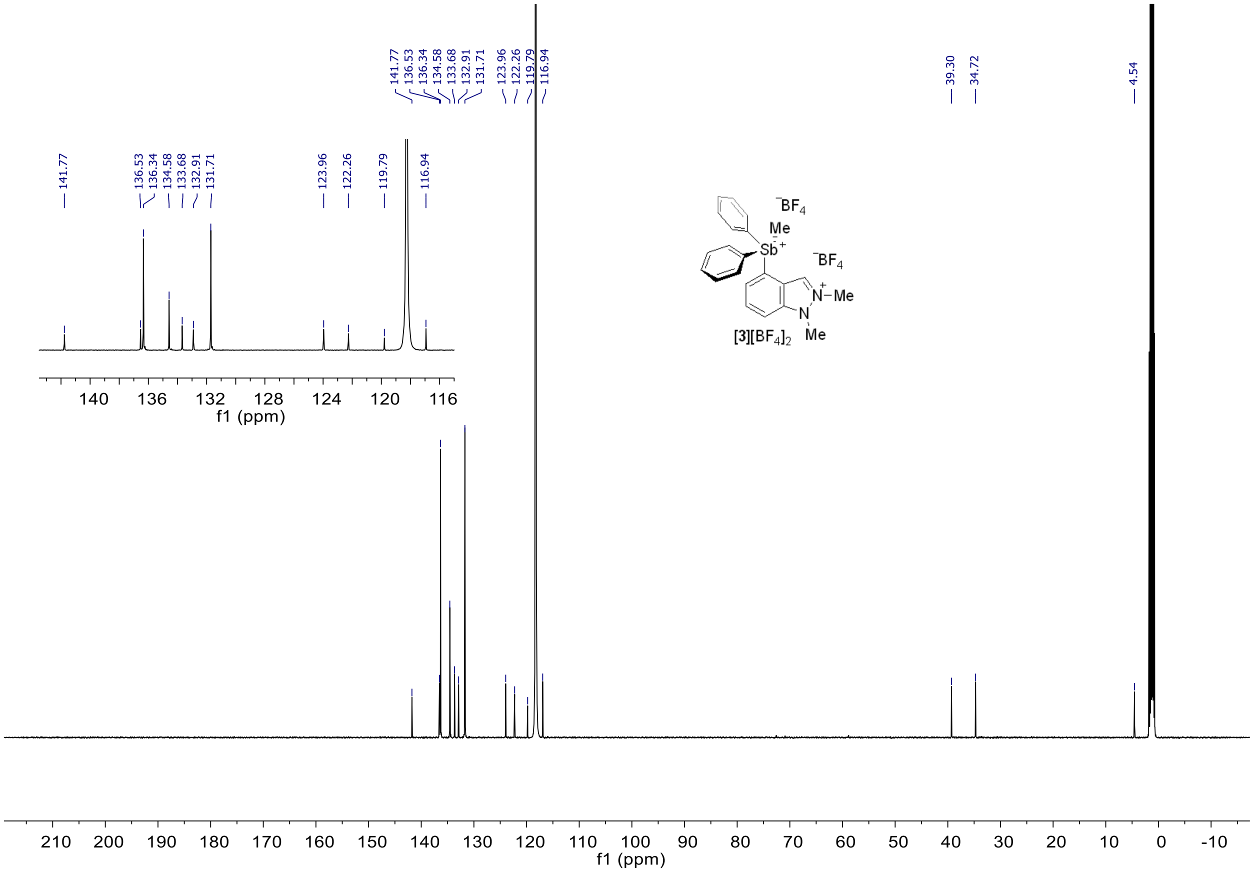

Figure S7. $^{13}C\{^{1}H\}$ NMR spectrum of [**3**][BF_4]$_2$in CD_3CN.The solvent peak is truncated.

NMR spectra for compound 4

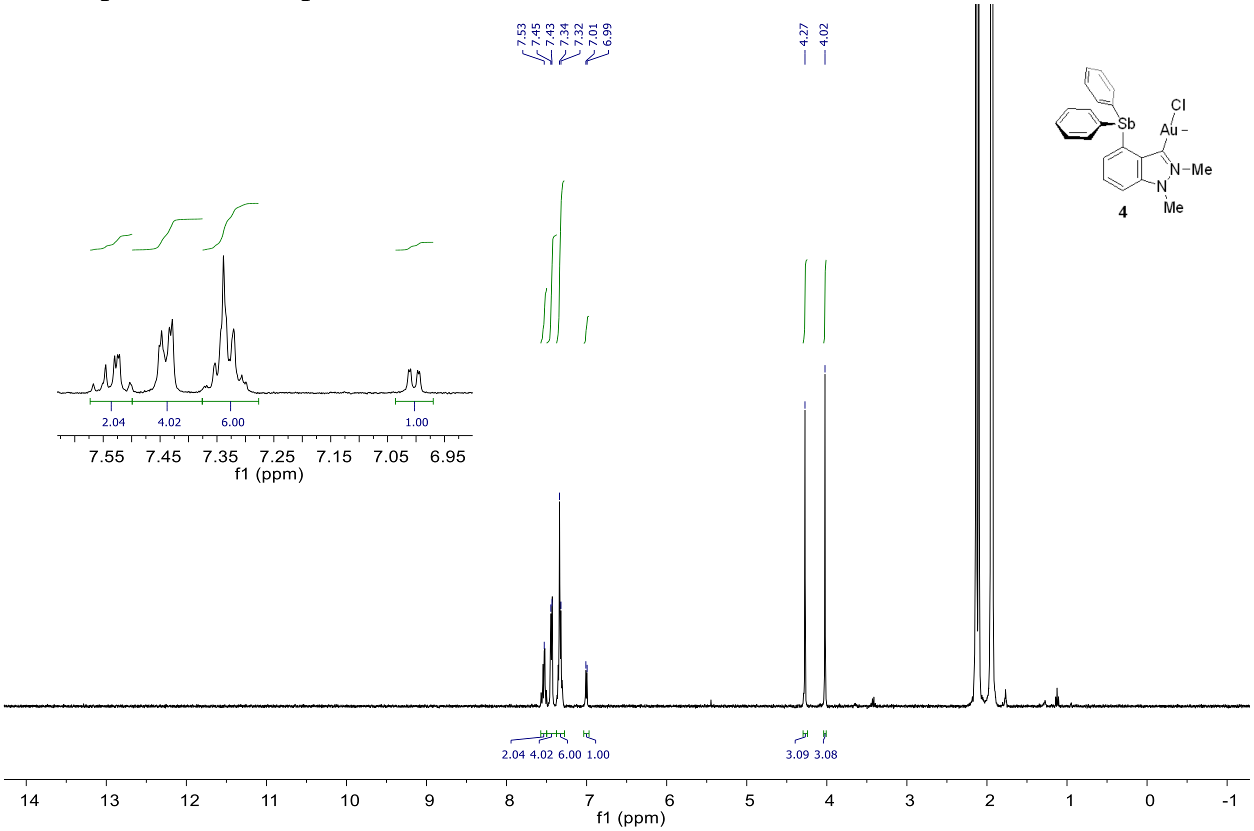

Figure S8. ^{1}H NMR spectrum of **4** in CD_3CN. The solvent peak is truncated.

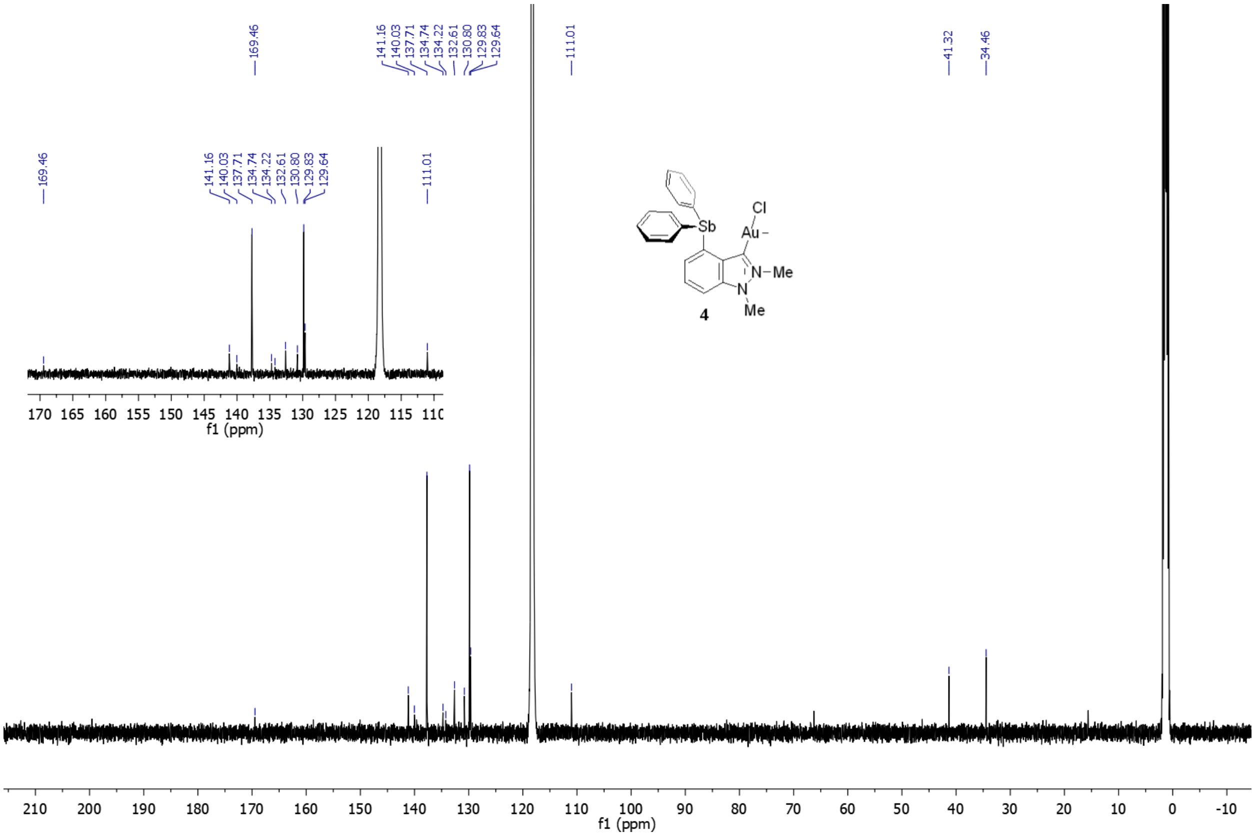

Figure S9. $^{13}C\{^{1}H\}$NMR spectrum of **4** in CD_3CN. The solvent peak is truncated.

NMR spectra for compound 5

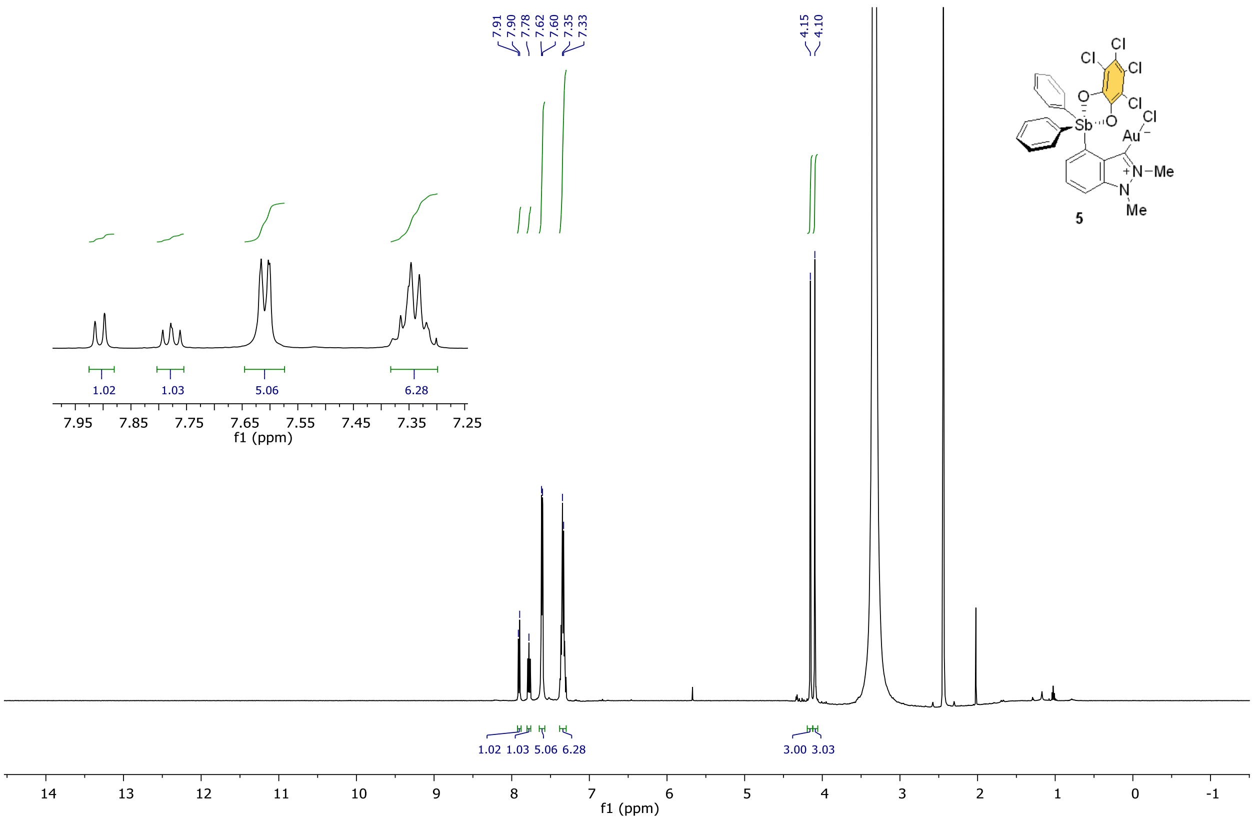

Figure S10. ^{1}H NMR spectrum of **5** in DMSO-d_6. The solvent peak is truncated.

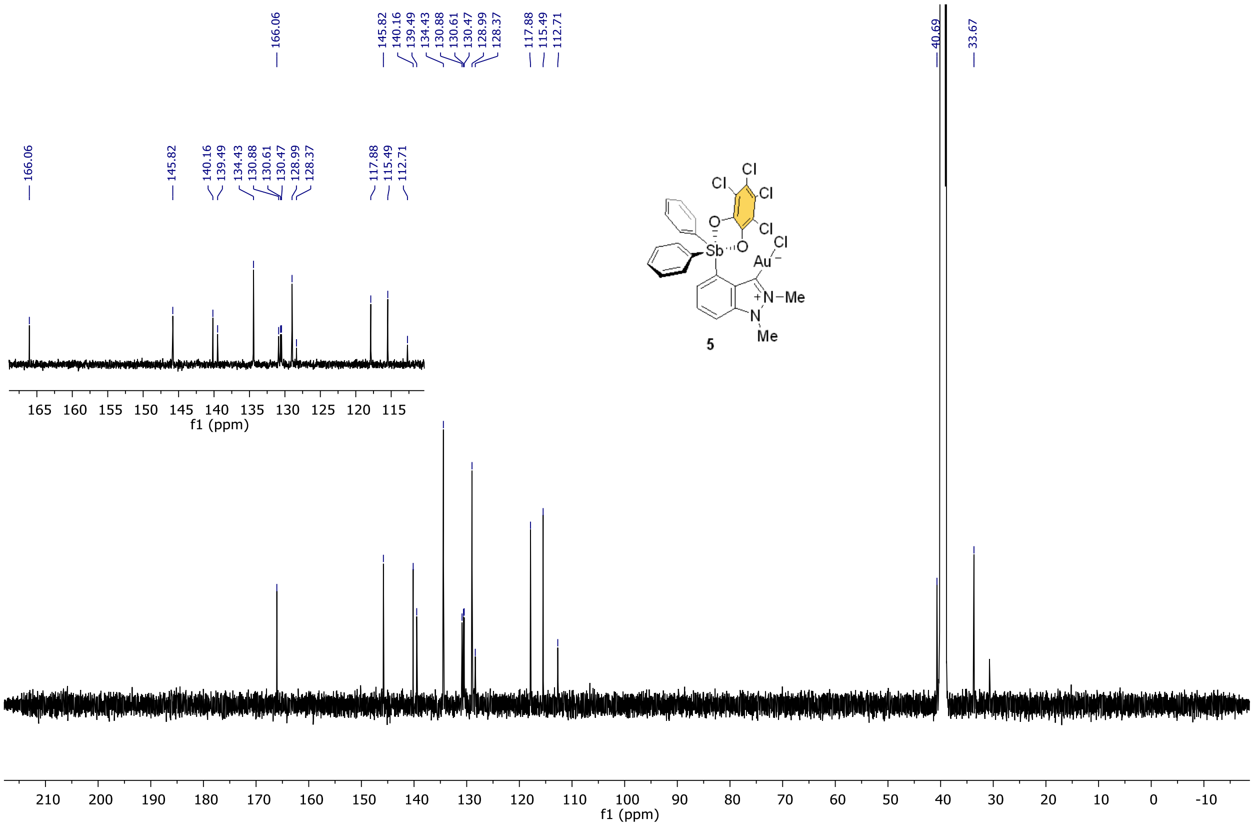

Figure S11. $^{13}C\{^1H\}$ NMR spectrum of **5** in DMSO-d_6. The solvent peak is truncated.

NMR Spectra for Catalytic Studies

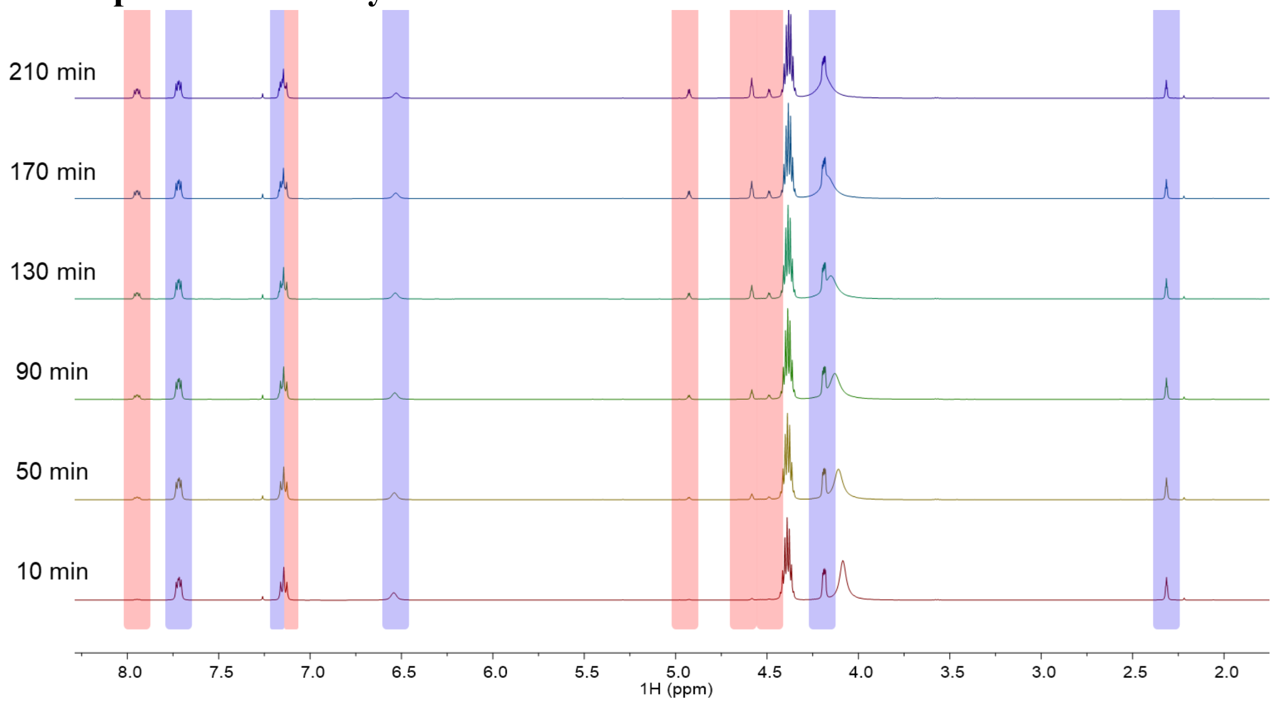

Figure S12. Stacked ^{1}H NMR spectra (499.41 MHz, $CDCl_3$) collected *in situ* during the cyclization of propargyl amide **6** (blue highlight) into **7** (red highlight) in the presence of **4** as the catalyst and HFIP as an additive.

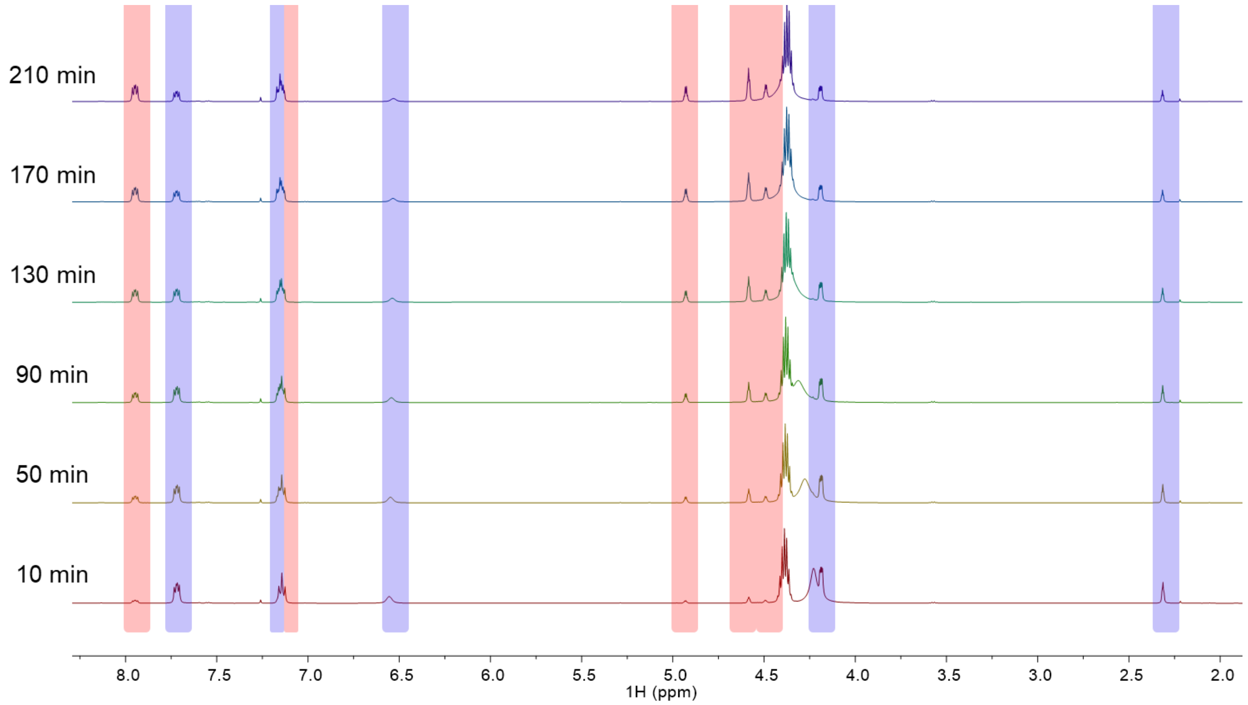

Figure S13. Stacked ^{1}H NMR spectra (499.41 MHz, $CDCl_3$) collected *in situ* during the cyclization of propargyl amide **6** (blue highlight) into **7** (red highlight) in the presence of 5 as the catalyst and HFIP as an additive.

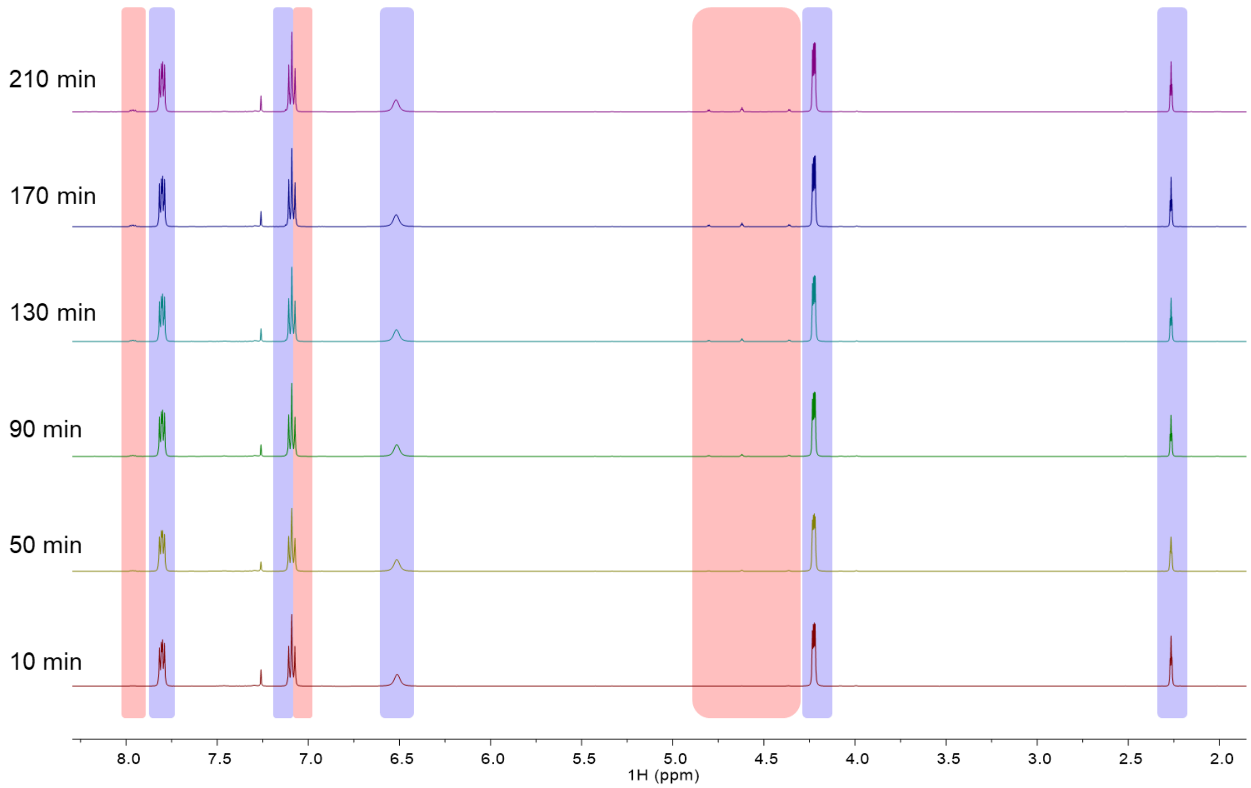

Figure S14. Stacked ^{1}H NMR spectra (499.41 MHz, $CDCl_3$) collected *in situ* during the cyclization of propargyl amide **6** (blue highlight) into **7** (red highlight) in the presence of **4** as the catalyst.

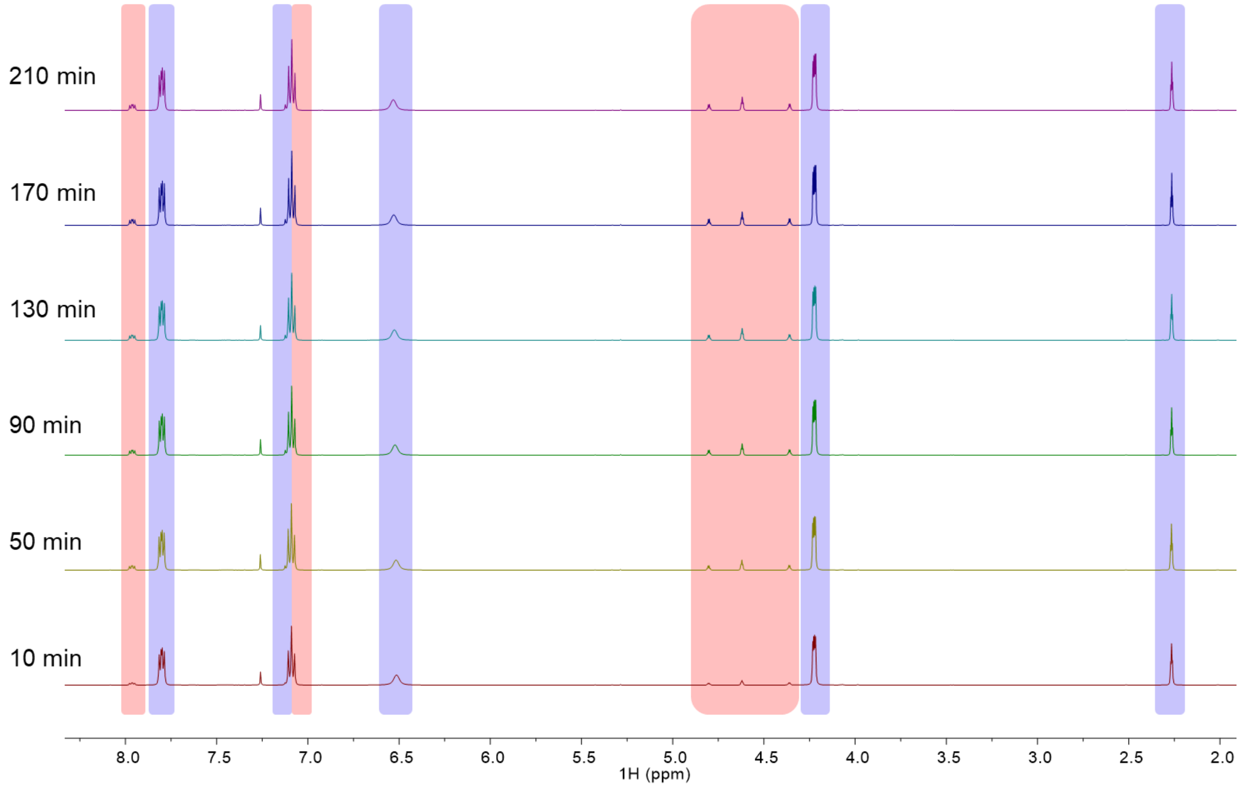

Figure S15. Stacked ^{1}H NMR spectra (499.41 MHz, $CDCl_3$) collected *in situ* during the cyclization of propargyl amide **6** (blue highlight) into **7** (red highlight) in the presence of **5** as the catalyst.

Computational details

Natural Bond Orbital (NBO) Analysis

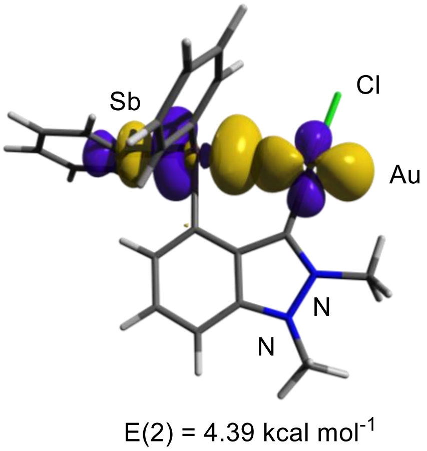

Figure S16. NBO orbitals involved in the lp(Au) → σ*(Sb-C) donor-acceptor interaction (isovalue = 0.05) in **4**.

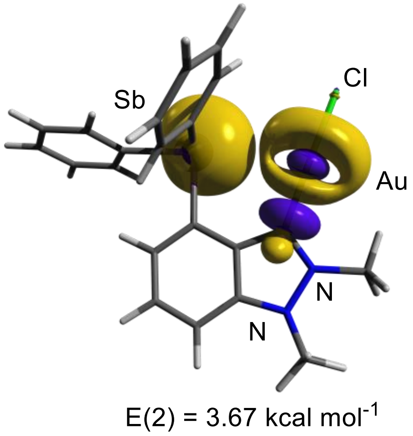

Figure S17. NBO orbitals involved in the lp(Sb) → σ*(Au-C) donor-acceptor interaction (isovalue = 0.05) in **4**.

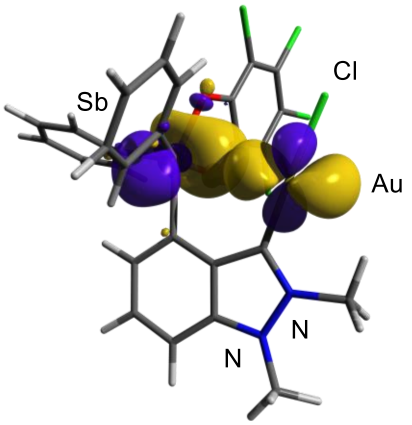

E(2) = 3.00 kcal mol^{-1}

Figure S18. NBO orbitals involved in the lp(Au) → lv(Sb) donor-acceptor interaction (isovalue = 0.05) in **5**.

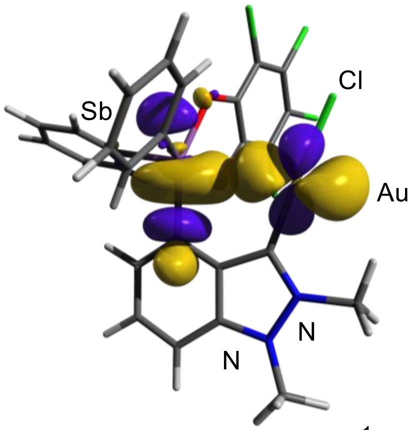

E(2) = 1.20 kcal mol^{-1}

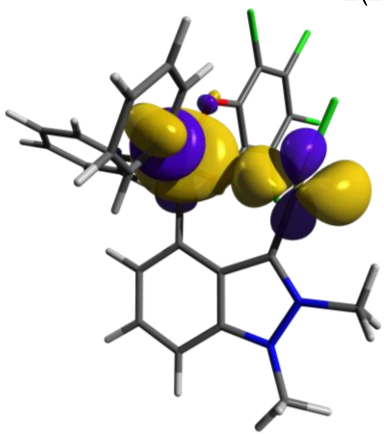

E(2) = 1.05 kcal mol^{-1}

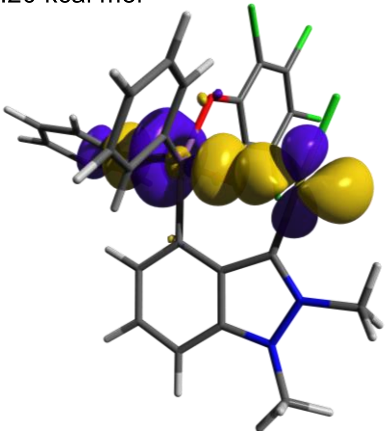

E(2) = 2.88 kcal mol^{-1}

Figure S19. All NBO orbitals involved in the lp(Au) → σ*(Sb-C) donor-acceptor interaction (isovalue = 0.05) in **5**.

Electrostatic potential maps

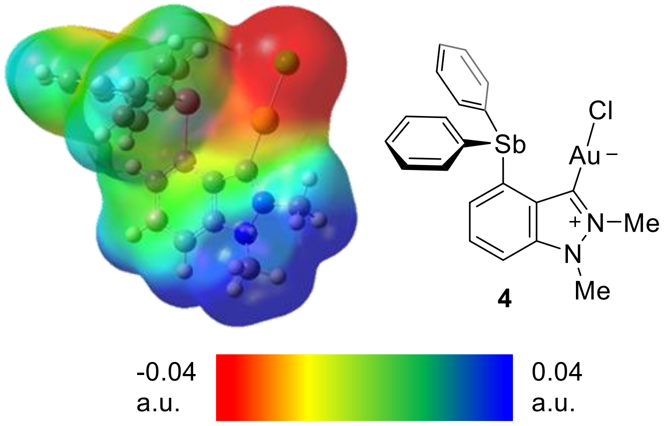

Figure S20. ESP map of **4**. All ESP maps are drawn with an isosurface value of 0.001.

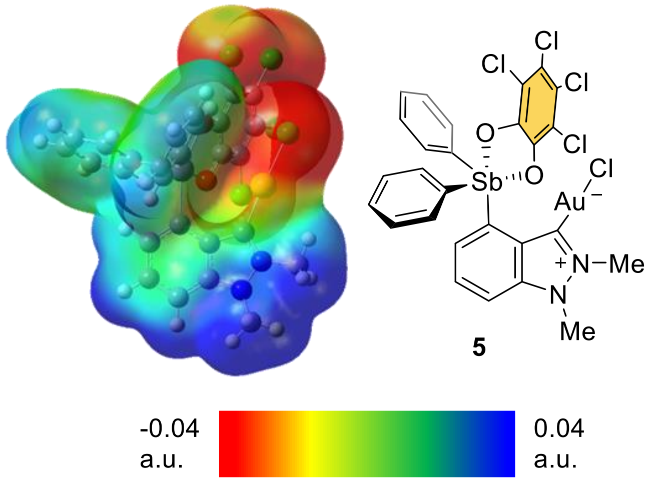

Figure S21. ESP map of **5**. All ESP maps are drawn with an isosurface value of 0.001.
